# Canine Rabies: A Looming Threat to Public Health

**DOI:** 10.3390/ani1040326

**Published:** 2011-09-26

**Authors:** Sigfrido Burgos-Cáceres

**Affiliations:** Food and Agriculture Organization of the United Nations, Viale delle Terme di Caracalla, Building C, Room 506, Rome, 00100, Italy; E-Mail: sigfrido.burgos@fao.org; Tel.: +39-06-570-53953

**Keywords:** canines, rabies, public health, zoonoses

## Abstract

**Simple Summary:**

This review is guided by three questions: What is canine rabies? Why is it a looming threat to public health? Why should we care about canine rabies being a public health threat? It seeks to answer these questions and notes that canine rabies is viral zoonosis with dogs being the major vectors. The disease is a looming threat to public health because rabid dogs bite humans, resulting in thousands of deaths every year. We should care about this evolving situation because, in general, rabies is a neglected disease for which there are vaccines, preventive measures, post-exposure prophylaxis, and control protocols.

**Abstract:**

Rabies is an acute, fatal viral disease that infects domestic and wild animals and is transmissible to humans. Worldwide, rabies kills over 55,000 people every year. The domestic dog plays a pivotal role in rabies transmission. Domestic dogs are not only part of our daily lives but also of our immediate surroundings, and this is reflected in the rise in pet dog ownership in developed and developing countries. This is important given that more frequent exposures and interactions at the animal-human interface increases the likelihood of contracting zoonotic diseases of companion animals. Despite existing vaccines and post-exposure prophylactic treatment, rabies remains a neglected disease that is poorly controlled throughout much of the developing world, particularly Africa and Asia, where most human rabies deaths occur. It is believed that with sustained international commitments, global elimination of rabies from domestic dog populations, the most dangerous vector to humans, is a realistic goal.

## 1. Canine Rabies

Rabies is an acute fatal viral illness of the central nervous system. It is also a viral zoonosis and dogs are the major vectors [1,2]. The rabies virus is a bullet-shaped, enveloped, RNA virus, 180 by 70 nm, of the *Lyssavirus* genus within the Rhabdovirus family. The helical nucleocapsid (N) is composed of a single-stranded negative-sense RNA genome and an RNA-dependent RNA polymerase enclosed in a matrix (M) protein covered by a lipid bilayer envelope containing knoblike glycoprotein (G) [2]. Transmission of rabies virus usually begins when infected saliva of a host (*i.e.*, a rabid dog) is passed to an uninfected organism (*i.e.*, a human being) [3]. To date, the most common mode of rabies virus transmission is through the bite and virus-containing saliva of an infected host [4]. Other transmission routes exist. These include contamination of mucous membranes (*i.e.*, eyes, nose, mouth), aerosol transmission, and corneal and organ transplantations. However, these alternative transmission routes have been rarely documented [5-8].

In animals, hyperexcitability, autonomic dysfunction, and aerophobia are characteristic of encephalitic rabies. The paralytic form is characterized by flaccid paralysis in the bitten limb, which ascends symmetrically or asymmetrically. In humans, the first symptoms of rabies include listlessness, general weakness, bodily discomfort, fever, pains, or headache. These symptoms may last for a couple of days. Later, there may be a prickling or itching sensation at the site of bite, progressing within days to symptoms of cerebral dysfunction, agitation, anxiety, and confusion. As the disease progresses, the person may experience abnormal behavior, delirium, hallucinations, insomnia, and respiratory failure. Once symptoms develop, the disease is often fatal. In fact, rabies remains one of the most ancient and deadly of human infectious diseases [2,5,8].

The virus incubation period in dogs may vary from one week to several months and may be influenced by the site of infection and the virus dose and strain. Diagnosis by clinical signs alone is inadequate since many rabid dogs develop dumb rabies which can easily be overlooked and others die without showing signs of rabies. Rabies virus may be excreted in the saliva before clinical signs appear and may lead to infection of an unsuspecting and untreated bite victim. Dogs may recover from clinical rabies and may then intermittently excrete virus in the saliva [1,3]. Rabies virus has been isolated from the saliva a female dog (that had been inoculated with a rabies isolate from the saliva of an apparently healthy Ethiopian dog developed rabies but later recovered without supportive treatment) collected at 42, 169, and 305 days after recovery [4,6].

Rabies is considered a fulminating disease given that once the first clinical signs appear, there is no effective treatment. However, a ray of hope emerged in 2004 with the report of a single patient recovering from rabies after aggressive, innovative treatment. Regrettably, this case was not clearly reproduced and the identification of targets for antiviral treatment in cases of rabies infection remains a major challenge [9]. Post-exposure prophylaxis consists of prompt and thorough wound cleansing and immunization with modern cell culture vaccines, together with administration of rabies immunoglobulin to those individuals who have not previously received pre-exposure prophylaxis [7,10]. On this specific topic, it is worth noting that historical reviews on the treatments and prevention of human rabies from ancient times up to the present have been undertaken [11,12].

## 2. Canine Rabies in the Context of Emerging and Reemerging Diseases

After 125 years of vaccination, rabies is still both a neglected disease and reemerging zoonosis [13-15]. It is now widely acknowledged that emerging and reemerging zoonotic disease events have heightened worldwide public awareness of the multidimensional linkages between domestic and wild animals, livestock production, and global public health [16,17].

There are many factors contributing to the emergence, reemergence, and intensification of zoonotic diseases. These include economic factors (*i.e.*, higher demand for animal foods, developing technologies, increased international travel, cross-border trade, novel agricultural, and industrial applications), social and cultural factors (*i.e.*, food habits, religious practices, lack of adequate health care, changes in human behavior, and farming practices), human and animal demographical factors (*i.e.*, ageing populations in developed countries, urbanization, population growth, availability of new hosts, and movement of animals into new landscapes), environmental factors (*i.e.*, global climate change, lack of adequate sanitation, and land use practices that result in human contact with previously remote habitats) and evolutionary factors (*i.e.*, microbial adaptations, enhanced infectiveness, and pathogen changes), to mention a few [18-20].

That most of the historical emerging diseases are intricately associated with very unique patterns of common determinants (*i.e.*, demographic, economic, environmental, *etc.*) suggests that an increasingly complex modern world will probably provide increasing opportunities for disease emergence and reemergence. Indeed, for centuries, emerging and remerging infections have remained among the principal challenges to human survival and a fundamental challenge to the existence and wellbeing of societies all around the world [21,22].

It would be wrong, however, to pin down diseases only to seemingly verifiable facts. Even if capable of infecting a different host species, pathogens are usually, although not always, significantly less infectious to them. This is referred to as the species barrier, and it can be substantial, implying that much higher doses are required to infect the new host. For example, the dose of rabies virus from foxes required to infect dogs and cats has been shown experimentally to be up to a million times greater than that required to infect other foxes [23,24].

A significant anthropogenic-dependent variable is dog care, disease knowledge, and leisurely practices. To give one example, a study to assess the knowledge and perceptions of dog-associated zoonoses in Brazos County, Texas, USA, demonstrated that many of the people surveyed lacked knowledge about dog-associated zoonotic diseases, which could seriously impact their health and the health of their families. Only 85 percent of respondents stated that they would seek emergency treatment if they believed that they may have been exposed to rabies, and only 59 percent of respondents were aware that exposure to rabies without treatment could lead to death [25].

These anthropogenic-dependent variables are critically important given that exposures at the animal-human interface are a key risk factor in canine rabies. On this subject, researchers at the U.S. National Center for Injury Prevention and Control argue that the dog bite problem should be re-conceptualized as a preventable epidemic. They claim that breed-specific approaches to the control of dog bites do not necessarily address the issue that many breeds are involved in the problem (not only dog breeds perceived as aggressive) and that most of the factors contributing to dog bites are related to the level of responsibility exercised by dog owners. To this end, in an effort to prevent dog bite-related deaths and injuries, recommendations advanced include stronger animal control laws, better resources for enforcement of these laws, better reporting of dog bites, and public education about responsible dog ownership and dog bite prevention [26,27].

In relation to public communication and education about disease prevention and control, an important development aiding to raise awareness on zoonotic diseases is fast communication technologies, leading to a growing number of independent health information brokers. For instance, internet search engines provide a wealth of information on rabies and other diseases, as well as incorporating features to detect onsets of seasonal flu epidemics—which not only matched official surveillance data but did so in advance. Today there is a widening range of outlets discussing health and diseases, and the internet, it appears, is democratizing health information; but its unverifiable character facilitates misleading others and being misled, thus warranting caution [20,28].

## 3. Lifestyles, Urbanization, and Pet Dog Ownership

A congeries of processes collectively labeled ‘modern globalization’ is impacting the way human beings conceptualize, deal, and interact with diseases [29,30]. One of these associated processes is urbanization. Rising urbanization is increasing the presence of traditional pets in households [31,32]. Many of these residences are in multi-building apartment complexes or condominiums where contact rates between people and dogs are increased, especially in playgrounds, leisure areas, and dog parks. Dogs are not only part of our daily lives and our immediate surroundings, but also have become substitutes for childbearing and child care to the point that owners allow dogs access to all rooms in an apartment or a house [33]. The rise in pet ownership is reflected in the amount of pet dogs in developed and developing nations. For example, the estimated pet dog population in the People's Republic of China is 100 to 200 million [34], in France is 8 million [32], in Taiwan is 2.5 million [35], in the Netherlands is 2 million [32], in the United Kingdom is 7 to 8 million [36], and in the United States is 60 million [32]. Overall, according to one estimate, the current world population of domestic dogs may be as high as 500 million, of which a substantial proportion is poorly supervised or free-roaming [37].

## 4. A Looming Threat to Public Health

Rabies remains a looming threat to public health in developing and transitioning countries, and the indigenous threat of rabies continues in developed countries because of wildlife reservoirs [38]. Both intergovernmental organizations and individual researchers estimate that rabies causes the deaths of over 55,000 persons every year, and this is known to be a conservative estimate. Most countries do not have the capacity for laboratory confirmation of rabies cases, and most suspected rabies victims do not die in hospital, so rabies is underreported [39,40]. In addition to human death tolls and mounting healthcare costs related to rabies, there are numerous other linkages between diseases, health, socioeconomics, and international affairs that should also be considered [41,42]. Because of the broad geographic spread of rabies in the world, the following subsections will focus on canine rabies in specific continents or regions. The purpose of this is to disaggregate the data and information into recognizable categories, and also to ease the exposition and explanation of content.

### Africa:

African countries are considered to be at particular risks of rabies infections. In fact, the World Health Organization (WHO) notes that more than 95% of human deaths occur in Africa and Asia [39]. Rabies has been, and continues to be, a public health threat in Angola [43,44], Southern Africa [44], Tanzania [45], and Zambia [46], among other countries. Here are a couple of examples: In South Africa, canine rabies has commonly been associated with the Eastern and Southern border areas in Mpumalanga Province, and, within this province, the Nkomazi District in the East has been most affected [47], as well as in the Limpopo Province [48]. Frequent outbreak reports throughout the country are probably an indication of inadequacy in the control of the disease at local levels. Moreover, the success and opportunism of rabies in Southern Africa is a reflection of the emergence and radiation of rabies in new host species and locales throughout the larger continent as a whole [49]. In Kenya, ever since rabies was first confirmed in 1912, the disease has largely existed in varying degrees of occurrence with dogs being the principal reservoirs for rabies. Over the years an enzootic pattern covering most parts of Kenya emerged, thus posing threats to neighbors [50,51]. One of the most acute problems is that, for most African countries, rabies prevention and treatment are costly and the necessary resources often scarce or inadequate. Controlling rabies in dogs, the main agents of spread, will therefore emerge as an important part of any rabies eradication program.

### Australia:

At the moment, Australia is free of canine rabies. This is partly explained because all imported animals are subject to strict quarantine requirements, including vaccination for dogs and cats from all affected countries. It is believed that the country's poorly controlled canine population, its indigenous fauna, and the casual Australian lifestyle would make a rabies outbreak difficult to control. In the past, dogs played pivotal roles in rabies. Three decades ago, in a survey of animal bites in Canberra, out of a total of 800 mammalian bites, 81 percent were due to dogs and cats [52]. In is interesting to note that until 1987, Australia had recorded only one case of travel-acquired rabies. Later, in 1990, an extreme case of long-incubation rabies was diagnosed in a 10-year-old girl of Vietnamese origin in whom rabies developed after she had lived continuously in Australia for almost five years [53,54]. While these cases are unusual, the evidence suggests that it can happen.

### Europe:

While rabies in domestic animals and wild carnivores has become extremely rare in Western Europe [55], the world witnessed the reemergence of rabies in some regions of Europe (Central and Eastern) that were previously designated rabies-free, which demonstrates the need for continual vigilance and the adoption of strict control measures for extended time periods. Despite the significant advances that have been made during the 20th century in reducing the burden of rabies, especially in Central and Eastern Europe, the disease remains endemic in many countries, largely as a result of financial limitations and a poor veterinary infrastructure [56,57]. For example, rabies has been endemic in Lithuania for decades, with wildlife cases principally reported in red foxes and raccoons from 1986 to 1996 [58]. Later, in the same country, a study from 1990 to 2000 reported that cases of rabies among foxes and raccoons had increased significantly [59]. In rabies situation reports, others have commented that an unfavorable situation remains mainly in the Baltic and nearby Southeastern countries [60], especially in Romania, Bulgaria, and Turkey [61]. In view of this situation, a number of Western European countries have voiced concerns on the threat of reintroduction of rabies virus. One of these is the United Kingdom, where risks from rabies exist on several levels. Based on assessments, scholars have positively commented on current UK government policy in light of a European call to harmonize rabies legislation across all Europe [62].

### Eurasia:

Without question, rabies in Eurasia is essentially a public health issue. Human rabies of canine origin has continued unabated for centuries in Eastern Eurasia, despite the Pasteur treatment and subsequent improvements of rabies post-exposure prophylaxis and novel biological products. In this sense, canine rabies, which is the main source of human contamination, remains practically uncontrolled. In these sub-regions three main rabies cycles are presently established: in dogs, in wild carnivores, and in insectivorous bats. Because of the strong barrier that exists between species-adapted rabies viruses and various potential hosts, these cycles are quite independent [63,64].

For instance, in Sanliurfa, Turkey, a study was launched to determine the level of knowledge of rabies transmission and control among physicians practicing in healthcare centers. The study found that while 96.4 percent of the physicians correctly indicated that cats and dogs can transmit the disease, the fact that foxes also have a role in transmission was known by only 48.8 percent [65]. The varying degrees of knowledge about common viral zoonoses reflect the need for continuous medical education at local, municipal, and provincial levels. Also, based on the peculiar conditions of the region, the authors noted that to control rabies, the issue must be dealt with locally, through both economic and social means [65]. In these regions, dogs are the major animal reservoirs, with wildlife maintaining recurrent cycles of infection as new viral etiological agents continue to emerge. Because nearly all human rabies cases in Eurasia are related directly to animal bites, primary disease prevention thus requires minimization of suspected exposures. Also, pre-exposure vaccination should occur in selected population groups at high risk of occupational exposure. Canine rabies elimination is the key towards ultimate reduction of disease burdens in Eurasia [66].

### South America:

Between 2006 and 2008, the largest urban rabies outbreak was reported in a Colombian city (Santa Marta) caused by a number of rabid dogs. The human health response was unprecedented; but, despite the existence of efficient rabies vaccines, the control of the outbreak was achieved 20 months after the first rabies case in dogs, and 14 months after the initiation of the first mass vaccination of animals [67]. Another study was carried out on canine rabies in Colombia to describe its tendency and explore the factors associated with its incidence. This study found that 10 variables were associated with the presence of canine rabies: an urban population, immunization coverage, a lack of a cold chain for vaccines, a lack of participation in surveillance committees, the lack of an epidemiological map, the unavailability of a rabies diagnosis laboratory, the absence of trained human resources, the absence of a zoonosis center for observing dogs, comparative analysis between monthly and semester basis data, and the percentage of people displaced by internal violence [68]. In Brazil, a study was launched to investigate the evolutionary history of dog rabies virus in the country, and concluded that the movement of rabid dogs, along with human activities since the 19th century, promoted the introduction and expansion of dog rabies virus in Brazil [69]. In Bolivia, the government issued a regulation for rabies control in 2005 owing to increases in the prevalence of dog and human rabies cases. In Santa Cruz de la Sierra, Bolivia, where dog rabies was endemic, an investigation on dog rabies vaccination coverage and risk factors associated with dogs being unvaccinated against rabies found that almost two-thirds of dogs were allowed to roam freely in the streets, parks, and yards throughout the day, with the majority of these dogs doing so unvaccinated [70]. From a historical perspective, it is worth noting that dog rabies had never been recorded in South America before European colonization [69], but today domestic dogs remain the most significant species for viral transmission, responsible for millions of suspect human exposures.

### South Asia:

There are some recent assessments of the burden of human rabies in South Asia [71-73]. For instance, in Eastern Bhutan, major outbreak of rabies in dogs and other domestic animals occurred between May 2005 and November 2007. This disease event resulted in one human and 256 domestic animal fatalities. It is believed that high densities and movements of free-roaming dogs might have been responsible for the rapid spread and persistence of the infection [74]. Later, in the first six months of 2008, rabies reemerged in the Chhukha district of Southwestern Bhutan [71].

In Northern India, an epidemiological study of 177 human rabies patients admitted to various hospitals in Amritsar city, Punjab, revealed that dogs were the source of exposure in 97.3 percent of cases and they were all suspected of having rabies. A history of second or third degree bites existed in all the cases [75]. Similar results were reported in a local hospital in Ahmedabad, Gujarat, in Eastern India [76]. The capital city, too, reports numerous cases of rabies due to dog bites. For instance, in an analysis of human rabies cases in Delhi the animal bites involved were largely of dogs, followed by jackals, cats, monkeys, and mongooses. Also, the male-female ratio was 4:1 probably suggesting that higher exposure to outside activities or field work is an important risk factor [73].

### Southeast Asia:

Rabies is a recurrent public health concern in Southeast Asia [77]. Thailand, for example, is a country visited by adventurers, off-the-beaten-path travelers, and backpackers every year [78]. In the first years of the 1990s, foreign travelers of multiple nationalities who had sojourned in Thailand for a little over two weeks were asked about potential exposure to rabies during their stays. Of the 1,882 travelers surveyed, 24 had been bitten and 167 had been licked by dogs [79]. Much later, in 2008, a survey of 870 foreign backpackers in their mid-twenties who visited Thailand's capital found that 31 had been licked by a dog [80]. Moreover, in a survey of exposure to rabies in humans, 48 out of 296 foreign aid workers and missionaries from Norway that traveled abroad were recommended post-exposure vaccination. Of these, 66 percent had either been licked by or had cared for a suspected rabid animal [81]. Moreover, rabies is also a concern to military officers.

In Vietnam, despite the accessibility to vaccines for both animals and humans, rabies remains a problem in many areas of the country. While the number of rabies deaths decreased by 90 percent from 1994 to 2003, the number of rabies deaths increased from 2004 to 2007 [82]. Later, a study to determine the molecular epidemiology of rabies virus in Vietnam revealed that Vietnamese and Thai rabies viruses are closely related and might have originated from a common ancestor [83].

In Cambodia, the rabies burden is largely underestimated because patients with encephalitis following dog bites are rarely hospitalized and die at home. Data from 2007 indicated that the estimated rabies-related mortality exceeded that of malaria and that of dengue. To make matters worse, free access to post-exposure prophylaxis is only sufficient for Phnom Penh residents [84].

In East Asia, too, rabies is emerging as a serious public health issue [34,85,86]. China has witnessed an increasing incidence of rabies in recent years and the number of deaths ranked first among the 39 notifiable infectious diseases [85]. A group of Chinese scientist ventured into exploring the possible origin, phylogenetic relationships, and evolutionary dynamics of Asian rabies viruses through examination of 200 complete nucleoprotein gene sequences from RABV isolates in the region. Their analyses demonstrated that China appears to be the prime source of Asian rabies viruses [86]. If there is a common theme among all of the above cases is that the epidemic of rabies is showing a rising trend in East, South, and Southeast Asia, as well as in Africa and the Americas.

## 5. Exposures and Interactions at the Animal-Human Interface

The first published estimate of a global disability-adjusted life year (DALY) score for rabies indicates that the disease exerts a considerable public health impact, exceeding other prominent diseases that currently achieve a higher priority for disease control [87]. This may be partly explained by exposures and interactions between humans and animals in urban, periurban, and rural settings. For instance, researchers conducted a matched case-control study to better define the risks associated with pets at both the household and individual levels. Their findings suggest that dogs may facilitate the transfer of pathogens and vectors into the home. Activities with close extended contacts with dogs may increase the risk of plague, rabies, and other infections [88]. Another factor to consider in animal-human exposures and interactions is dog bites. Dogs that are less restricted in their interactions with humans are at elevated risk for biting; however, links between interaction and dog bites in one cultural setting might not exist in another [89]. A case review of dog bites notes that children and elders are usual victims of unsuspecting rabid dogs [90]. Also, as already noted in some cases in Thailand, dog licks may pose a health risk. In many countries around the world, be it developed or developing, the licking of humans by dogs that are rabid or suspected to be rabid poses a major risk to human health [32]. For example, there is a report of a patient who developed meningitis due to *Pasteurella multocida* transmitted by a dog that frequently licked his ear [91].

It would be a mistake to think that exposures and interactions with dogs are limited to outdoors, playgrounds, dog parks, or even households. As dogs are widely accepted in other social settings, they are more prevalent in more locations too. For example, dogs are probably the most widely used animals in pet therapy and seem to have a very positive therapeutic effect. They also appear to potentially transmit the greatest number of zoonotic diseases [92]. In a study that measured for the first time the prevalence of zoonotic organisms among dogs that actively visit hospitals as part of an organized program, researchers identified many concerns regarding the potential for carriage of zoonotic pathogens (including rabies) by dogs involved with visitations. While it is widely recognized that visitation of hospitalized people by dogs is becoming commonplace, more attention needs to be given to the potential health risks of introducing dogs to healthcare settings [93].

Leading scientists and researchers around the world have been trying to understand the global temporal and spatial patterns of animal diseases through an array of instruments and cutting-edge molecular technologies to track the genetic makeup of infectious pathogens. If there is a commonly shared outlook among experts it is that novel zoonotic diseases will continue to emerge [94]. It is with this conviction that governments around the world are moving forward with strong plans to prevent and control diseases with high public health impacts. The prevention of human rabies is accomplished by controlling rabies in domestic and wild animals, including the use of vaccination programs [95]. Thankfully, technology has advanced rapidly since modern human rabies vaccines were developed over 40 years ago. However, the usability of human rabies vaccines is hampered by high cost, complicated vaccination regimens, and lack of compliance, especially in areas of Africa and Asia where human rabies infections are endemic. It is believed that a single-dose vaccine would greatly benefit efforts to combat this global health threat. However, a single-dose vaccine based on current inactivated vaccines does not appear feasible and other approaches are needed [96]. To this end, rabies DNA vaccines have shown good efficacy in preventing rabies in some experimental animal models; regrettably, their performance in post-exposure treatments has been less impressive. In view of lackluster outcomes, the development of current DNA vaccines to rabies for use in humans is, at current times, not entirely appropriate [97].

On the topic of vaccines and their applicability, it worth noting that, in the United States, a move to provide dogs and cats with a triennial rabies vaccine was opposed by hundreds in the veterinary community, some concerned that its implementation would be followed by a decrease in rabies vaccination rates. A study attempting to document a decrease in rabies vaccination rates found positive changes (increases) in rabies vaccination rates following migration from one to three-year vaccination intervals [98]. Lastly, experts and physicians continue to remind dog owners, victims of dog bites, nurses, and veterinary professionals that there are certain risk factors that must be considered when deciding on post-exposure treatment for rabies. These include (a) age of victim, (b) site of bites, (c) severity of wound, and (d) the state of dog's health [99,100]. The next section will share reflections on what to expect of canine rabies in the second decade of the 21st century.

## 6. Finding Guidance and Perspective: Looking Ahead and Beyond

Many of the current approaches to disease prevention and control emphasize transmission disruption. Whilst critically important, this approach in itself does not address the root causes of disease incidence. One of the options to effectively deal with the latter is to more emphatically address and tackle the drivers of zoonotic diseases [101]. Understanding the factors for emergence and reemergence of high-impact infectious diseases requires a holistic perspective that incorporates socio-cultural as well as physical, chemical, and biological dimensions of our planet's systems. The notion of bio-complexity captures this depth and richness, and most importantly, the interactions of humans with natural ecosystems [102]. In the case of rabies, one of the drivers is the increasing interactions of animals (*i.e.*, pets and free-roaming dogs) with the pathogen in multiple environments and settings. The elimination of rabies viruses from the animal reservoir constitutes an investment in preventing rabies in humans. Veterinary public health practice has demonstrated that a reduction of canine rabies correlates with a substantial decrease in human rabies cases. If rabies is eliminated from domestic animals and wildlife, the incidence of rabies in mankind will also be controlled.

Animal rabies can be controlled by proper induction of herd immunity, humane removal of stray animals, promotion of responsible pet ownership through education, and enactment of leash laws, among many other measures [1,3,5,7]. Others believe that the control of rabies largely depends on the prevention of infection of dogs by vaccination in endemic areas and the control of their movement, including measures of quarantine and vaccination [103]. In Western Africa, for example, where rabies runs rampant, the building of veterinary capacity through post-secondary education has been proposed as a viable measure that could uphold public health in countries such as Benin, Burkina Faso, Côte d'Ivoire, Senegal, and Sierra Leone [104]. In Matongo, Tanzania (East Africa), locals in the region are encouraged to have their domestic dogs vaccinated against rabies to prevent cross-species transmission as part of a campaign to reduce the risk to public health [105]. The few positive experiences in Africa show that community-based active surveillance provides a potentially cost-effective strategy for greatly improving estimates of rabies incidence and to enhance epidemiological studies geared to inform veterinary and policy decision making [49-51].

Moreover, in 2008, rabies experts from 14 francophone African countries met in Côte d'Ivoire to discuss the disease that for long has been affecting their continent. They presented the situation in their respective countries, acknowledging the lack of rabies awareness among the population, health care workers, and health authorities. They fully recognized that infrastructure for the management of rabies exposure is scarce, accessible and modern vaccines are limited, and immunoglobulins are lacking in most of their countries. They defined as a priority the need to have reliable figures on the disease burden, which is necessary for informed decision making and priority setting, and for applying for foreign aid in controlling rabies in urban and rural settings. This meeting ended with the establishment of the Africa Rabies Expert Bureau (AfroREB). In view of these efforts, other continents and regions can learn from what is being done in Africa to establish their own rabies prevention and control centers. In the end, it is the necessity of implementation and maintenance of rabies control strategies that is so strongly underlined for minimizing human risks and threats.

With regards to rabies control in agricultural and natural ecosystems, alternative paths and routes have been proposed. A number of suggestions have been advanced to monitor rabies in bats, but the rare cases of rabies transmission directly to humans from bats arise because rabies changes a bat's behavior so that it does encounter and bite humans, which a healthy bat (other than a vampire bat) would never do [106,107]. Another alternative path is wildlife vaccines. Vaccinations to reduce diseases among wildlife species becomes a contested alternative given a number of complex issues associated with economics and practicality, scientific debate regarding effectiveness, conservation ecology, and public perception. For over 34 years, it has been shown that oral-route vaccination could generate protective immune responses in domestic and wild animals (dogs and foxes). This was the finding that led to the success of rabies wildlife vaccine usage, and it should continue [108].

Some commentators believe that with sustained international commitments, global elimination of rabies from domestic dog populations, the most dangerous vector to humans, is a realistic goal [109]. Overall, the promotion of responsible dog ownership combined with effective vaccination and sterilization of owned dogs would have to be implemented, and regular vaccination of dogs continued. If the medical infrastructure is strengthened by educating more healthcare and veterinary workers and improving the availability of safe and effective biological products, especially animal vaccines and human rabies post-exposure prophylaxis, the world would be able to drastically reduce human rabies cases. Also, the current rabies programs could be improved by better supervision, improving interaction between authorities, increasing rabies awareness, and altering urban planning and development to balance the interaction between humans and animals. We must recognize that decades of extraordinary scientific and technological progress coupled with mass-reaching information technologies now grant collective confidence that development and diffusion of best practices, lessons learned, and continuing innovation can advance our world much further in better management of zoonotic diseases that arise at the animal-human interface and also now offers other cardinal directions for a healthy and prosperous environment for all [16]. Although a major focus of the recent efforts towards global health governance schemes will be on zoonotic infectious diseases [110], there are critical areas within this contemporary initiative which small companion animals (*i.e.*, cats and dogs) should play a significant role. The availability of the canine genomes and the development of microarray genomic screening tools provide the world with the unprecedented ability to explore the basis of canine diseases that so closely mimic those that occur in man [111].

## 7. Personal Reflections: Awareness-Raising, Prevention Strategies, and Mitigation Efforts

Education is an important element in the prevention and control of rabies. Teaching about rabies and post-bite measures to students in primary schools and the first levels of secondary schools may prove beneficial in the long-term. The assumption is that by teaching students, they will carry this knowledge home to their families and friends, and in turn disseminate information to a wider community. The belief is that teaching children about rabies provides a solid foundation for reducing risks of contracting rabies and thus aim for a rabies-free future. In comparison to adults, it is easier for children to learn new information through already familiar activities at school. The incorporation of rabies education into school curricula is not simply a one-time educational event, but rather a sustained effort as the information becomes disseminated throughout countries and regions each year without the need for recurrent monetary resources. To date, there are anecdotal reports of very positive experiences coming out from schools in the Philippines and Sri Lanka on the introduction of rabies related educational information into their regular school curriculums.

Prevention strategies are critical to better manage rabies in endemic areas. Vaccinating domestic dogs can substantially reduce the numbers of canine rabies and, most importantly, human rabies cases. But, for this to happen, government-funded registration and licensing of dogs should be made compulsory and enforced at city or town level. Overall, dog population management and the promotion of responsible dog ownership combined with vaccination and sterilization of owned dogs in rural and urban areas would have to be implemented, and regular vaccination of dogs continued. Also, existing rabies prevention programs could be improved by better supervision, improving interaction between authorities, increasing rabies awareness, and altering urban planning and city development to balance the interaction between humans and animals.

In many locations around the world, taskforces on rabies prevention and control have been established to bring together both animal and human health professionals and scientists to review current policy frameworks and foster a multidisciplinary approach across national agencies involved in rabies surveillance and control. Any mitigation effort must keep in mind that the most important reservoir of rabies is the domestic dog, and through canine vaccination and controlling dog populations, countries can dramatically reduce rabies exposure to humans.
